# Radiographic identification and description of the equine middle conchal sinus (ethmoidal sinus): Implications for diagnosing middle conchal sinusitis

**DOI:** 10.1002/evj.70198

**Published:** 2026-06-08

**Authors:** J.‐B. Pavard, A. Young, M. Robert, D. Verwilghen

**Affiliations:** ^1^ Goulburn Valley Equine Hospital Congupna Australia; ^2^ Equicare—Vétérinaires équins Chantilly France; ^3^ Alex Young Specialist Veterinary Imaging Australia; ^4^ CHVE de Livet Livarot‐Pays‐d'Auge France; ^5^ Melbourne Veterinary School, Faculty of Science University of Melbourne Werribee Australia

**Keywords:** diagnostic imaging, horse, middle conchal sinus, radiographic identification, sinusitis

## Abstract

**Background:**

The middle conchal sinus (MCS) (ethmoidal sinus) remains one of the least understood equine paranasal compartments. Despite recognition of its involvement in sinonasal disease, disorders of the MCS often remain underdiagnosed in the absence of computed tomographic (CT) imaging due to limited anatomical understanding and challenges with radiographic visualisation.

**Objectives:**

The objectives of the study were (1) to characterise the radiographic features and anatomical location of the MCS in horses and (2) to illustrate the practical relevance of the anatomical and radiographic findings in two clinical cases of MCS sinusitis.

**Study Design:**

Descriptive anatomical and imaging study.

**Methods:**

Six equine cadaver heads representing different age groups underwent standard radiographic projections before and after sagittal sectioning and placement of wire markers outlining the MCS. Radiographs were evaluated to identify and describe anatomical landmarks. Two clinical cases of MCS sinusitis confirmed by CT were used for comparative assessment.

**Results:**

The MCS was not readily identifiable on pre‐dissection radiographs. Post‐sectioning images revealed consistent latero‐lateral radiographic landmarks, notably its ovoid shape superimposed on the ethmoturbinates and its location dorsal to the third maxillary molar (maxillary Triadan 11's). Oblique projections allowed differentiation on which side was affected. On dorsoventral projections, the MCS was consistently superimposed on the middle nasal meatus, the nasal septum and the rostral margin of the ethmoturbinates, allowing tentative localisation in all specimens. The clinical cases had pathological changes at the predicted MCS site, correlating with cadaveric observations.

**Main Limitations:**

The main limitations of the study include the small number of specimens examined and that radiographic interpretation was performed by a non‐blinded reviewer.

**Conclusions:**

This study provides a reproducible radiographic reference for localising the MCS using radiography. Improved awareness of its location and presentation on radiographs will aid clinicians in diagnosing sinus disease involving this compartment, especially when CT imaging is unavailable.

## 摘要

研究背景:马中鼻甲窦(middle conchal sinus, MCS)是马副鼻窦系统中研究较少的解剖结构之一。尽管其在鼻窦疾病中的作用逐渐受到关注，但由于对其解剖结构认识不足以及常规X线影像显示困难，在缺乏计算机断层扫描(CT)的情况下，MCS相关疾病往往被低估或漏诊。

研究目的:本研究旨在:(1)描述马MCS的影像学特征及其解剖定位；(2)通过两例确诊的MCS鼻窦炎病例，阐明上述影像学与解剖学发现的临床意义。

研究设计:描述性解剖与影像学研究。

研究方法:选取6个不同年龄阶段的马头标本，在矢状面切开前后分别进行标准X线投照。通过放置金属标记线勾勒MCS边界，对影像进行评估，以识别及描述解剖学标志。本研究还纳入2例经CT确诊的MCS鼻窦炎临床病例进行对比分析。

研究结果:在解剖前的X线影像中，MCS难以清晰识别。经矢状切开及标记后，在正侧位(latero‐lateral)投照中，获得了可重复识别的影像学特征，MCS表现为卵圆形影像，叠加于筛骨鼻甲(ethmoid turbinates)之上，并位于上颌第三臼齿(Triadan 11)背侧。斜位投照有助于区分左右侧。在背腹位(dorsoventral)投照中，MCS持续叠加于中鼻道(middle nasal meatus)、鼻中隔(nasal septum)及筛骨鼻甲前缘，从而可在所有标本中实现大致定位。临床病例显示病变位置与推测的MCS解剖位置一致，与标本大体解剖观察结果相符。

主要局限性:本研究样本量较小，且影像学判读未采用盲法分析。

研究结论:本研究建立了一套基于常规x线影像技术的MCS定位参考方法，具有良好的可重复性。提高对其解剖位置及影像表现的认识，有助于临床，尤其在在缺乏CT条件下，提高对该部位窦腔疾病的诊断能力。

## 1 INTRODUCTION

The paranasal sinuses are clinically important in equine medicine as disease is common^1^ and diagnosis and treatment remain particularly challenging.^2^, ^3^ The complex anatomy of the different sinuses can be challenging to understand, yet this anatomical knowledge is crucial for managing these diseases.^1^, ^4^, ^5^, ^6^, ^7^

Developments in diagnostic imaging, and more particularly of multiplanar reconstruction of computed tomographic (CT) images has confirmed horses to have eight pairs of paranasal sinuses divided by the maxillary septum into two sinus complexes.^2^, ^4^, ^5^, ^6^ The rostral sinus complex is composed of two compartments: the rostral maxillary sinus (RMS) and the ventral conchal sinus (VCS). The caudal sinus complex comprises the other six compartments: the caudal maxillary sinus (CMS), the dorsal conchal sinus and the frontal sinus (collectively referred to as the concho‐frontal sinus) (CFS), the sphenoidal sinus, the palatine sinus (collectively named the sphenopalatine sinus—SPS) and the middle conchal sinus (MCS), also called ethmoidal sinus.

The CFS, RMS and CMS have long been considered as commonly involved in paranasal sinuses disease.^8^, ^9^ However, a better anatomical knowledge on sinonasal disease has generated new interest in less commonly described structures including the dorsal and ventral nasal conchal bullae,^10^ the SPS^11^ and the MCS.^12^ Although the MCS is a referenced compartment of the equine paranasal sinuses,^4^, ^5^, ^7^ this structure has only infrequently been identified to be affected by disease.^9^ Yet, its poor identification may result in under‐diagnosed disease.^2^, ^12^ The MCS is a small, poorly demarcated compartment of the paranasal sinuses, anatomically corresponding to the large spiral of the ethmoid,^13^ which remains inconsistently described and poorly represented in standard imaging.

The development of cross‐sectional diagnostic imaging modalities, such as CT, has benefited our spatial recognition of anatomical structures and allowed us to describe their relative position, relationships, and volumes in healthy horses in order to improve our understanding of their disorders.^4^, ^5^, ^6^, ^7^ However, due to its expense, CT imaging facilities are limited to referral equine practice. In contrast, radiography or endoscopy are readily available as first‐line diagnostic imaging modalities. Given the anatomical complexity of equine paranasal sinuses, the interpretation of two‐dimensional radiography is challenging and makes precise location of sinonasal structures difficult, especially for the little referenced sinuses like the MCS.

This study aims to characterise the radiographic features and anatomical positioning of the MCS to enhance understanding of its appearance on imaging and to illustrate the description with two clinical cases affected by MCS sinusitis.

## 2 MATERIALS AND METHODS

### 2.1 Cadaver specimens

The heads of six horses of three age groups (<6 years old: *n* = 3, 6–15 years old: *n* = 2, >15 years old: *n* = 1) of various breeds, humanely euthanised for reasons unrelated to the study and grossly free from sinus pathology, were collected after disarticulation at the atlanto‐occipital joint.

### 2.2 Radiographic protocol

The six heads were initially radiographically screened using standard latero‐lateral (LL), left latero30°dorsal‐right lateroventral oblique (L30°D‐LVO), right latero30°dorsal‐left lateroventral oblique (L30°D‐LVO) and dorsoventral (DV) projections centred over the maxillary 08–09 region to confirm the absence of abnormal radiographic changes possibly linked to sinus disease. Then, the heads were sectioned sagittally using a band saw and the two halves were separated. The nasal septum was partly cut and elevated without being completely excised to visualise the paranasal cavities. The right and left MCS were identified, and steel wires were placed along the external anatomical delimitations of both MCS delineating the rostro‐caudal and dorso‐ventral perimeters of their external walls. Two different diameters of steel wires were used to differentiate the right and left sides on radiographs, the smallest being used for the right side.

The two halves of the heads were joined and attached together with tape after having replaced the nasal septum in its original position. Six radiographic projections were taken including right and left LL projections, right and left latero30°dorsal‐lateroventral oblique projections, dorsoventral and ventrodorsal projections. As the actual location of the MCS was now identified, the focus of the beam was more accurately centred over the MCS which corresponded with approximately midway between the medial canthus of the eye and the ventral orbital rim on a line perpendicular to the facial crest. On dorsoventral projections, it was centred on the midline between the medial canthi of the eyes.

The acquisition of the images was performed with a portable generator (Atomoscope Through Ray TR9030 GEN2, Radincon, Australia) with a setting of 75 kV and 0.08 mAs/s and a wireless 35 × 43 cm x‐ray flat panel detector (Canon CXDI‐710C, Radincon, Australia).

The radiographic analysis was performed using VetRocketPacs (vetrocket, California, USA) image processing software that facilitated assessment of the size, shape and symmetry of the MCS for each head. The relative anatomical position of the two sinuses was assessed in relation to the maxillary cheek teeth and ethmoturbinates. Approximate measurements were made using lines drawn perpendicular to the horizontal plane at the level of the rostral and caudal limits of the MCS.

The various projections were reviewed by a board‐certified imaging specialist in order to identify the most suitable projection to allow the most complete anatomical characterisation of the MCS. The MCS was identified on the post‐dissection specimens with the wires in place and their locations described for each specimen on each of the different radiographic projections.

### 2.3 Clinical cases of MCS sinusitis

Two clinical cases of MCS sinusitis confirmed by CT were included for comparison with the cadaveric findings.

A 4‐year‐old racing Thoroughbred (TB) filly presented for unilateral purulent nasal discharge was diagnosed with 109 apical infection and chronic right paranasal sinusitis following CT of the head. The investigation identified involvement of the right MCS (Figure 1A).

**FIGURE 1:**
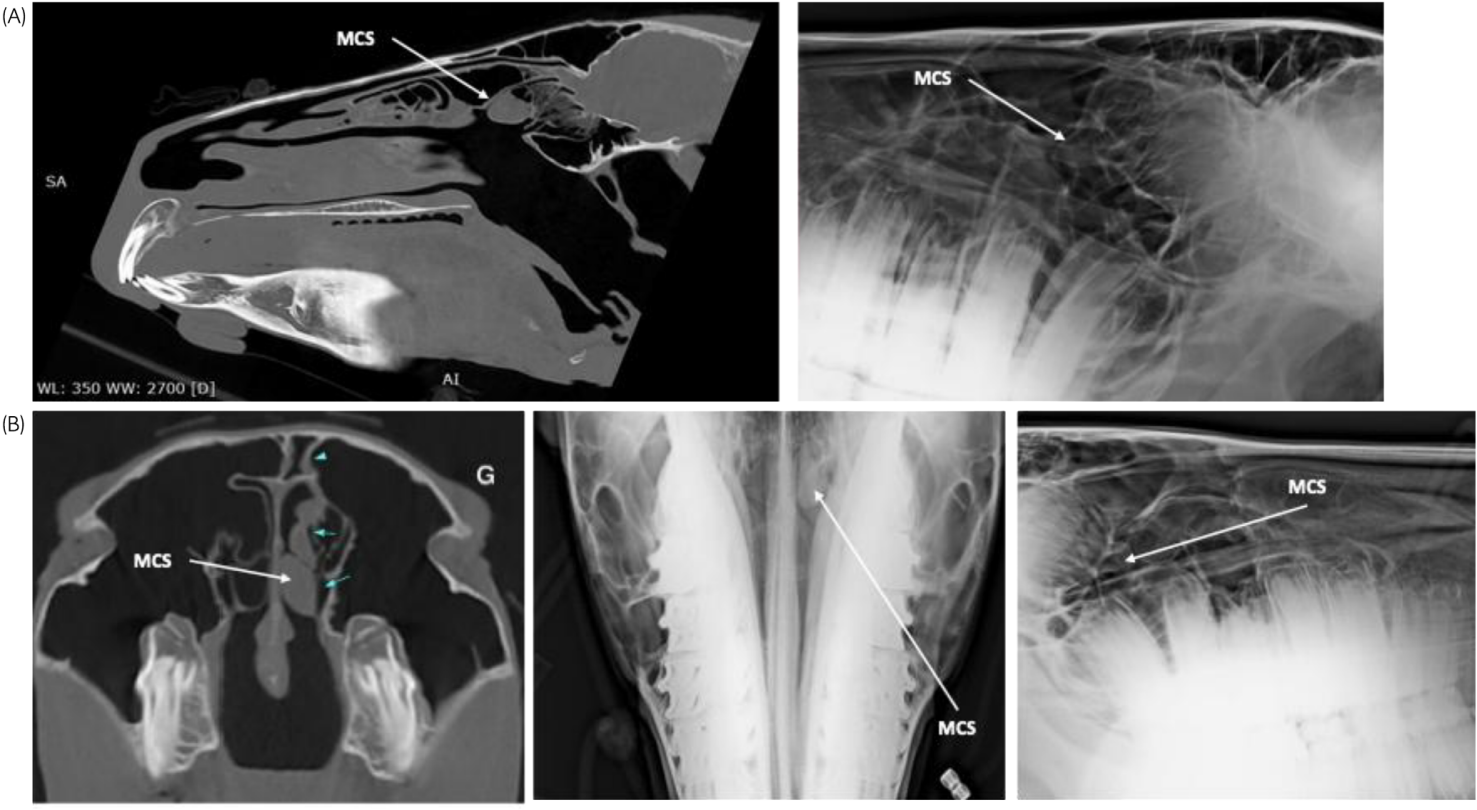
(A) Case 1 (4‐year‐old Thoroughbred, right‐sided sinusitis): Sagittal CT head image (left) showing right‐sided paranasal sinusitis with involvement of the MCS (white arrow). The MCS is also clearly visible on the latero‐lateral radiographic projection (right). (B) Case 2 (4‐year‐old Standardbred, left‐sided rhinosinusitis): Transverse head CT image (left) showing left‐sided rhinosinusitis with MCS involvement (white arrow). The MCS is more clearly visualised on the dorso‐ventral radiographic projection (middle) than on the latero‐lateral projection (right).

The second case was a 4‐year‐old Standardbred (SB) gelding presented for chronic unilateral nasal discharge and diagnosed with chronic left rhinosinusitis following head CT examination. This revealed no evidence of dental disease (considered to be the cause of the sinusitis), and a primary sinus infection was suspected, with imaging findings suggestive of a possible fungal process, although not confirmed. Involvement of the left MCS (Figure 1B) was identified on CT, with the presence of material consistent with fluid attenuation.

Radiographs from both clinical cases were collected as described above and the appearance and location of the MCS were compared with our findings in cadaver heads of horses without sinus disease.

## 3 RESULTS

### 3.1 Radiographs before sagittal sectioning and placement of markers

No sinus disease was observed on the pre‐dissection radiographs of the six equine cadaver heads. This set of radiographs did not allow for confident identification of the MCS (Figure 2).

**FIGURE 2:**
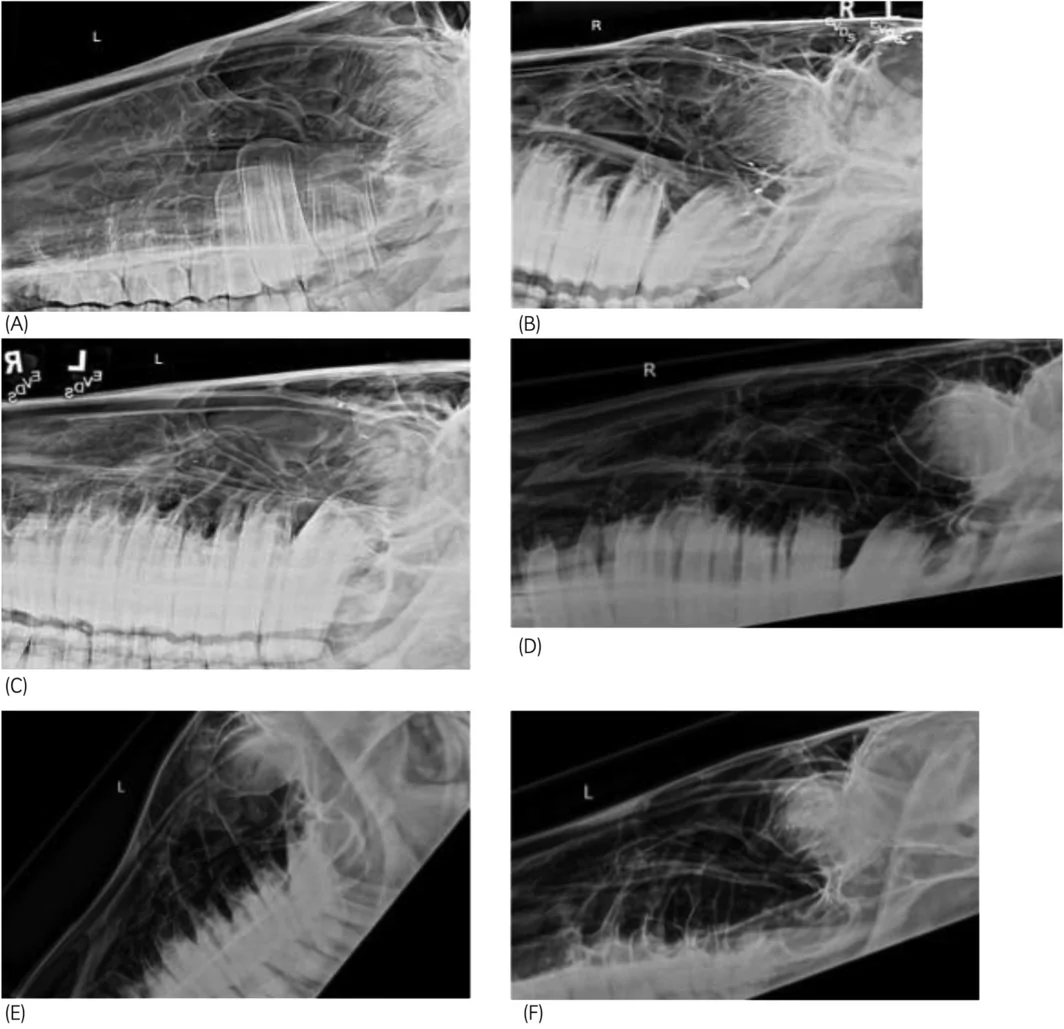
Latero‐lateral radiographic projections of six cadaver heads (A –F), obtained prior to dissection and arranged from the youngest to the oldest. Age categories were as follows: (A) <2 years; (B–D) 3–5 years; (E) 6–15 years; and (F) >15 years. These images show no abnormal radiographic changes in the paranasal sinuses and illustrate the difficulty in confidently identifying the MCS on standard radiographs.

### 3.2 Descriptive clinical anatomy of the middle conchal sinus

In all the sectioned half heads, the MCSs were identified in the caudo‐dorsal area of the nasal/paranasal cavity after excision of the nasal septum (Figure 3). The MCS forms the cavity of the middle nasal concha (MNC) just rostral to the ethmoturbinates, which are composed of the ethmoidal meati and conchae.

**FIGURE 3:**
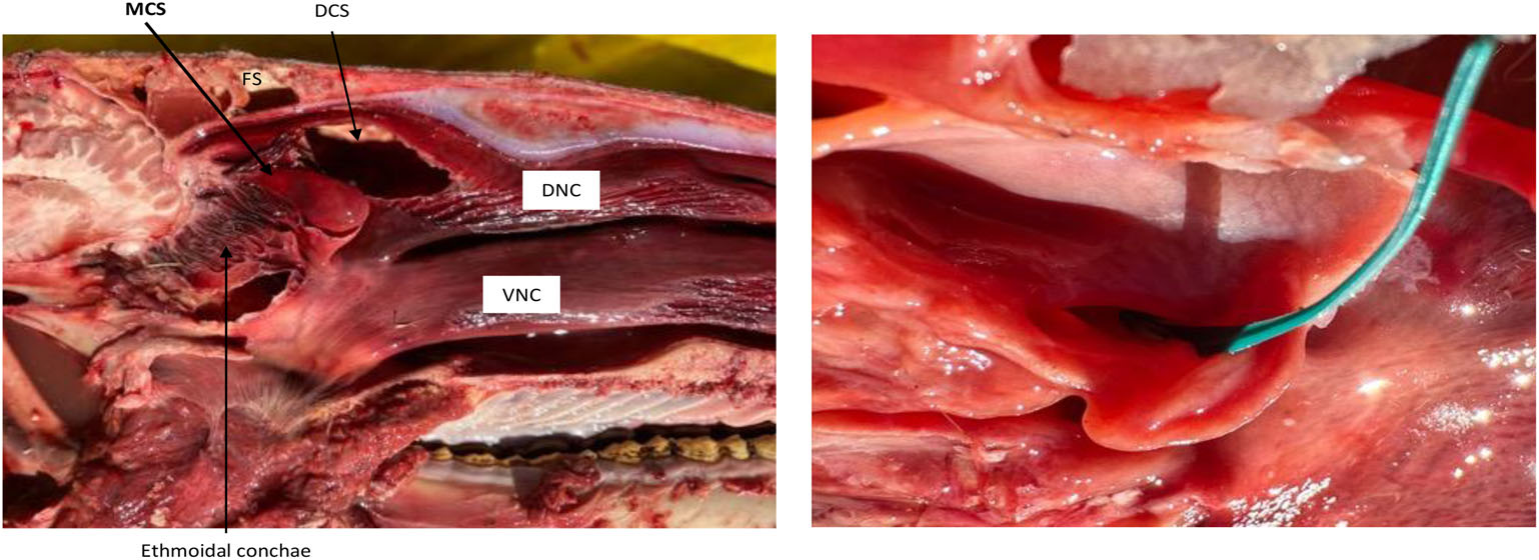
Left: Medial aspect of the left side of a cadaver equine head illustrating the location of the MCS after excision of the nasal septum. The dorsal conchal sinus (DCS), the frontal sinus (FS), the dorsal nasal concha (DNC) and the ventral nasal concha (VNC) are also identifiable. Right: Blue wire pointing toward the ostium, confirming drainage of the MCS into the CMS in the same head.

Partial removal of the medial wall of the MCS in the sectioned heads allowed identification of an orifice confirming drainage of the MCS into the CMS (Figure 3).

### 3.3 Identification of radiographic landmarks of the middle conchal sinus using metallic markers

Sagittal sections of the six heads allowed gross visualisation of the MCS which resulted in modification of the beam focus for subsequent post‐gross dissection radiographs.

### 3.4 LL projections

The radiographically identifiable ethmoturbinates were the most useful radiographic landmark for the MCS on LL projections. On these projections, the wire outlining the MCS was consistently superimposed on the rostroventral aspect of the radio‐opaque ethmoturbinates, approximately 45° between the rostral and ventral turbinate margins. The MCS was ovoid in shape with its long axis aligned in a rostroventral to caudodorsal orientation and was superimposed on approximately one third of the overall ethmoturbinates opacity (Figure 4).

**FIGURE 4:**
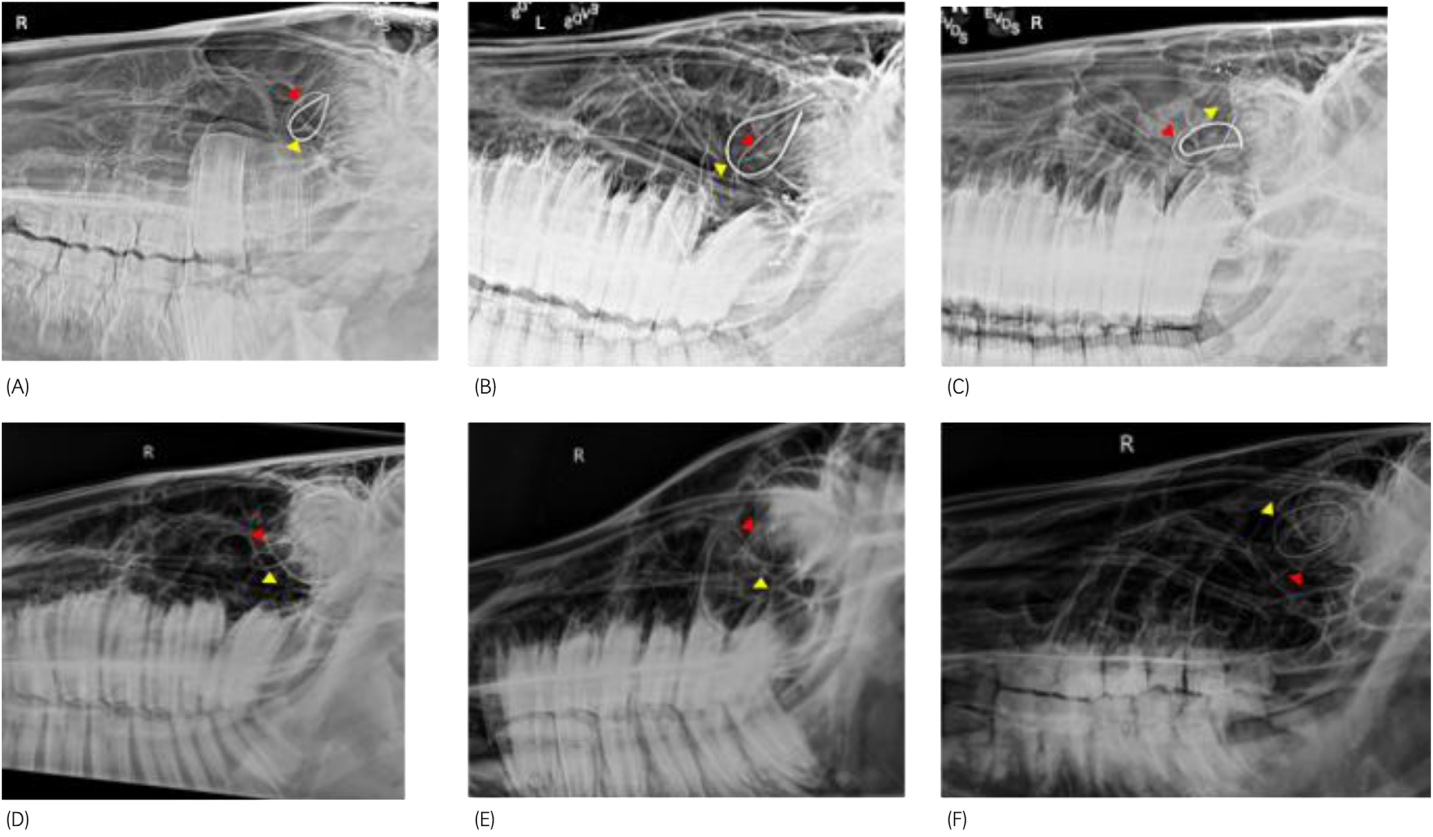
Latero‐lateral radiographic projections obtained after placement of metallic wires (the thinner diameter indicating the right side), outlining the right (red arrow) and left (yellow arrow) MCS in six cadaver equine heads (A–F). The MCS consistently appears in close association with the ethmoturbinates complex, providing a reliable anatomical landmark across all specimens. In contrast, variation in the position of the MCS relative to maxillary Triadan 11's was observed and is likely influenced by age. Age categories were as follows: (A) <2 years; (B–D) 3–5 years; (E) 6–15 years; and (F) >15 years.

The location of the MCS in relation to the cheek teeth varied between the different heads and appeared to be age‐related. In the youngest horse (approximately 2 years old), the MCS was located dorsal to the caudal aspect of maxillary Triadan 10's and just rostral to developing maxillary Triadan 11's, which was not yet clearly visualised on radiographs. In four of the six heads, the MCS was located dorsal to maxillary Triadan 11's. The only >15‐year‐old horse had multiple cheek teeth close to expiration, and the MCS was subsequently located caudal to the remaining maxillary Triadan 11's.

### 3.5 Latero30°dorsal‐lateroventral oblique projections

The MCS remained superimposed on the ethmoturbinates on the oblique projections and was unchanged in their rostro‐caudal relationship to maxillary Triadan 11's. There was a small difference in the dorsoventral location of the left versus right MCS on these projections, which was expected and attributable to the somewhat axial location of these structures and their proximity to the nasal septum (Figure 5). This dorsoventral separation of the MCSs allowed for determination of the left versus right MCS, which is essential for assessment of left vs. right sided sinonasal disease.

**FIGURE 5:**
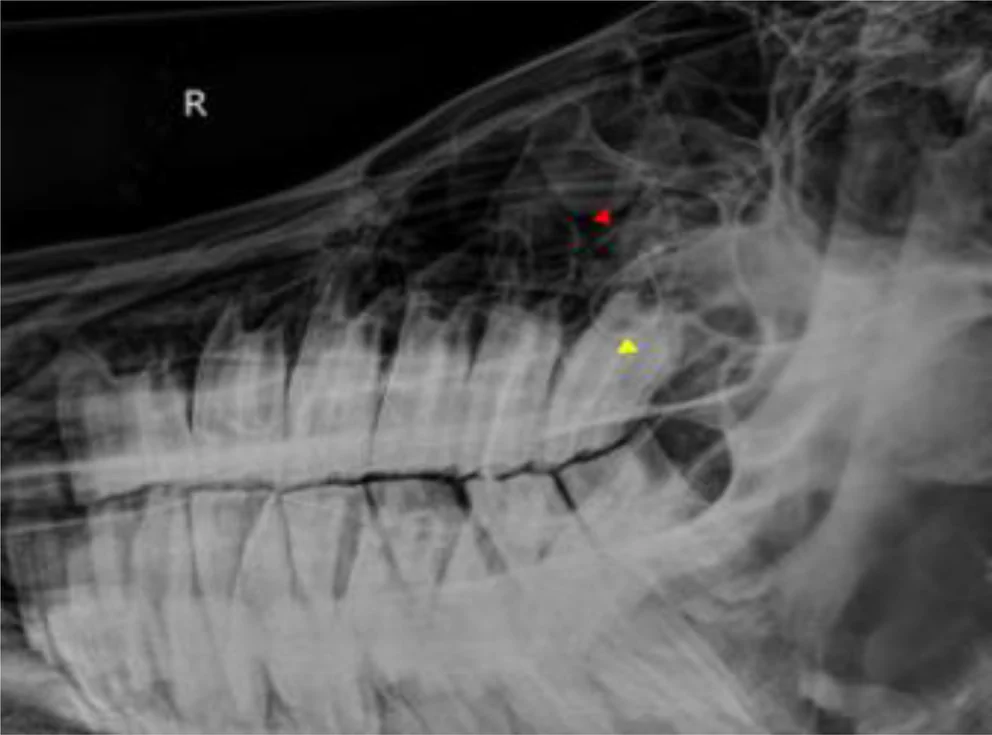
Left dorsal‐right ventral oblique projection after the placement of the wires (the thinner diameter indicating the right side) in a cadaver equine head allowing dorsoventral separation of the right (red arrow) versus left (yellow arrow) MCSs.

### 3.6 Dorsoventral and ventrodorsal projections

Each MCS was superimposed on rostral opacity of the ethmoturbinates, and on the linear gas lucency of the ipsilateral middle nasal meatus and the nasal septum. In the youngest horse (approximately 2 years old), the rostral border of the MCS was located at the level of the caudal roots of Triadan 110/210 on both dorsal‐ventral and ventro‐dorsal projections. In the four other horses aged <15 years, it was located at the level of the caudal roots of maxillary Triadan 11's. In contrast, in the only horse aged >15 years, the entire wire delineating each MCS was caudal to maxillary Triadan 11's. In all six heads, the MCS were identifiable superimposed on the middle nasal meati (Figure 6).

**FIGURE 6:**
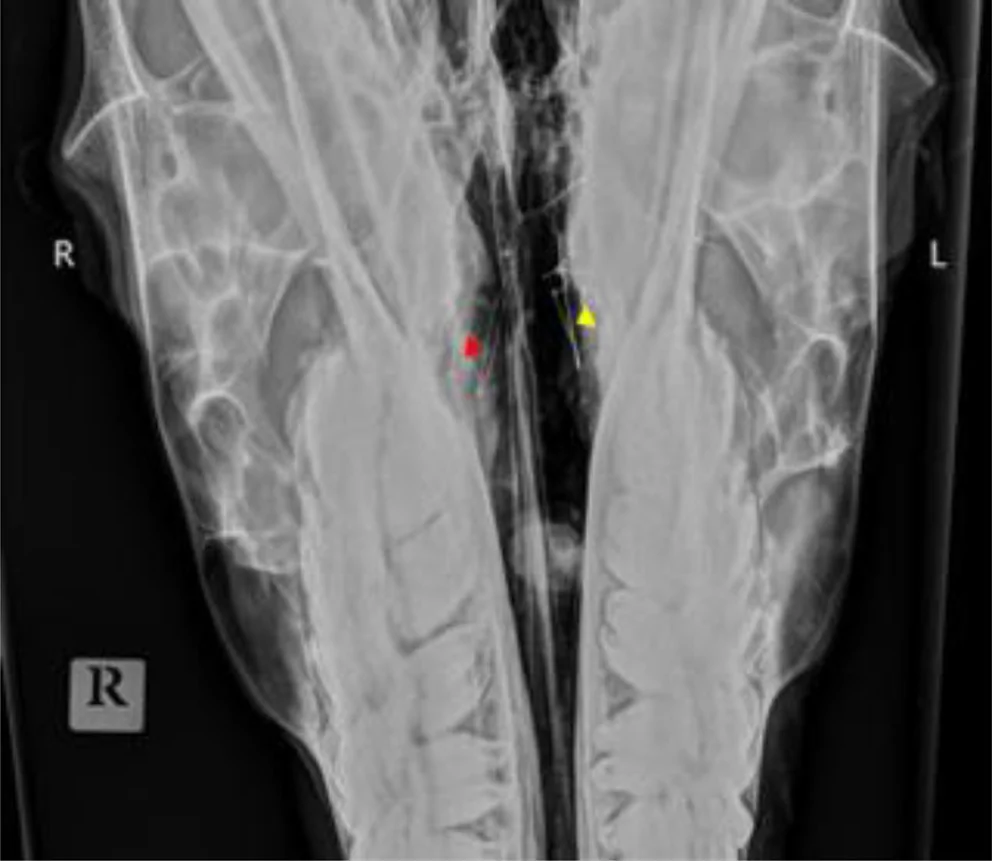
Dorso‐ventral radiographic projection after the placement of the wires (the thinner diameter indicating the right side) in a cadaver equine head allowing identification of the right (red arrow) and left (yellow arrow) MCSs.

### 3.7 Radiographic appearance of the MCS in the two clinical cases and comparison with the findings of non‐affected cadaver heads

On the LL projection centred on maxillary Triadan 10's/11's, the MCS was identifiable in both clinical cases. In the first case, the MCS contained some inspissated exudate, and it appeared as a marked soft‐tissue opacity, similar to that of the other affected sinuses, with a visible fluid line. In the second case, the MCS was also delineated, although with a less pronounced soft‐tissue opacity.

On the dorso‐ventral projection of the second case, the MCS was clearly identified, showing a marked soft‐tissue opacity (Figure 1B).

That allowed delineation of the MCS external walls, revealing an ovoid shape and a location consistent with the findings of the cadaveric study. The clinical radiographs corroborated the MCS anatomical relationship with the ethmoturbinates and maxillary Triadan 11's, consistent with observations in the horse heads older than 2 years and younger than 15 years in this study.

## 4 DISCUSSION

The MCS, also called ethmoidal sinus, has been identified as a cause of primary or secondary sinusitis.^3^, ^9^, ^14^ According to the study of Dixon et al.,^9^ the MCS and SPS were affected in 7% of sinus diseases. However, this involvement was noted by the authors as difficult to confirm in these patients. A more recent CT study of 300 equine sinusitis cases identified the MCS to be affected in 32% of cases.^14^ Despite the frequency of disease in this location, the MCS remains a little‐referenced sinus, rarely cited in scientific literature and often forgotten as a possible structure affected by sinus diseases.

The development of CT imaging has improved our anatomical knowledge of the MCS and facilitated better characterisation of its position and communication in relation to the other paranasal structures. Relative to the other sinuses of the caudal paranasal sinus complex, the MCS has the smallest volume with a mean of approximately 4 cm^3^.^5^ Other more recent CT‐based studies have provided additional insights into MCS volumetry and report similar overall volume ranges, while differing in specific findings. Liuti et al.^7^ reported that all sinus compartment volumes were positively associated with head volume and varied significantly with age, with mean MCS volumes increasing from approximately 2.4 cm^3^ in young horses (<6 years) to 4 cm^3^ in middle‐aged horses and 5.6 cm^3^ in older horses (>15 years). In contrast, Borowska et al.^4^ did not identify significant age‐related differences in MCS volume, reporting comparable values across age groups (3.2–3.9 cm^3^). In the present study, no obvious macroscopic differences in MCS size were observed between specimens of different ages. However, volumetric assessment was not performed, and precise differences may not be detectable without CT‐based analysis.

The MCS drains laterally into the CMS.^2^, ^5^, ^6^ This direct communication can allow a CMS sinusitis to cause secondary MCS infection, as is likely the case in our first clinical case. Here, a dental sinusitis (Triadan 109 infection) likely initially affected the CMS, and through adjacent communication, the infection subsequently spread to the MCS. However, the present study also suggests that the MCS may be involved in primary sinus disease not related to dental pathology. In our second clinical case, head CT examination confirmed involvement of the left MCS, associated with findings consistent with a chronic left rhinosinusitis. Although detailed CT findings are beyond the scope of this report, they included marked mucosal and osseous changes within the left nasal cavity and paranasal sinuses, with no dental‐related abnormalities identified, supporting a primary unilateral sinusitis with a suspected fungal component. In some cases of MCS infection, it is noteworthy that the MCS may appear reddened and oedematous on nasal endoscopy and may even develop a fistula into the nasal cavity.^9^ In our experience, similar endoscopic findings have also been observed in cases of sinusitis without CT confirmation, which is useful given that endoscopy is a widely accessible diagnostic tool compared to CT in equine practice. Moreover, based on the clinical experience of some of the authors, endoscopic laser fenestration and drainage of the MCS may be successfully performed in selected cases.

Initially, the present study sought to describe the normal radiographic appearance and location of the MCS in relation to identifiable landmarks in heads of horses without sinonasal disease. Although the approximate location of the MCS can be determined on a lateral‐lateral radiograph using the readily visible rostral margin of the radio‐opaque ethmoturbinates, the exact margins of the MCS could not be directly visualised radiographically prior to the placement of markers, and there were no well delineated radiolucent structures that could be accurately identified as the MCS. This may explain why the MCS is rarely described in sinusitis cases diagnosed radiographically.

As described previously, radiographs after the placement of markers showed common useful landmarks on LL projections in all six equine heads with the relation of MCS to ethmoturbinates complex position.

Although the MCS remained superimposed on the ethmoturbinates and was unchanged in its rostrocaudal relationship to maxillary Triadan 11's on L30°D‐LVO projections, it allowed separate identification of the right and left MCSs which is clinically important.

Ventro‐dorsal projections were performed to determine if they could allow a better identification of the MCS compared to dorso‐ventral projections. The variation did not seem to allow a substantial difference in identification, yet our positive contrast wire reference was relatively easy to visualise on both projections. The situation may be different in cases with naturally occurring disease. However, in the second clinical case reported in this study, the affected MCS was easily and clearly identifiable on the DV projection (Figure 1B), indicating that its infection can facilitate its visualisation.

The position of the MCS was also assessed in relation to the cheek teeth. In all six equine heads, it was located relatively close to the maxillary Triadan 11's roots. However, some variations in location and delimitation were observed between the six heads that were likely age‐related. Although age‐related rostral drift of the maxillary cheek teeth has been relatively under‐investigated in the literature, Liuti et al.^7^ quantified this phenomenon and reported that the maxillary Triadan 11's drifted rostrally by a mean of 2.48 cm at the caudal clinical crown and 2.83 cm at the apices relative to the orbit in old horses (>15 years old) compared to the young horses (<6 years old), a difference that was statistically significant. This observation is consistent with our findings: in younger horses, the MCS position appeared more cranial and closer to the maxillary roots compared to older horses (Figure 4). These results suggest that apparent cranial or caudal shifts in MCS position relative to the cheek teeth are primarily attributable to age‐related movement of the cheek teeth rather than changes in the MCS itself.

In the second phase of the study, we compared our findings with radiographs from two clinical cases of MCS sinusitis confirmed by CT. This comparison supported the diagnostic value of radiography, particularly on LL and DV projections, validating the anatomical landmarks identified in our sample. It also highlighted that radiographs can be sensitive in detecting MCS sinusitis as an ovoid soft‐tissue opacity—provided the clinician is familiar with optimal imaging techniques and can accurately localise the structure despite overlapping anatomy in the region.

To our knowledge, radiographic identification of MCS sinusitis remains rarely reported. This raises the question of whether diagnostic difficulty varies with clinical presentation, or whether poor knowledge of the radiographic appearance of the MCS is the primary barrier.

Importantly, it is not uncommon for secondary sinusitis to persist even after the primary cause, such as dental disease, has been resolved.^15^, ^16^ The continued presence of purulent material in sinus cavities—most notably the ventral conchal sinus and the ventral and dorsal conchal bullae—can result in ongoing nasal discharge. A similar mechanism may apply to the MCS, which has a narrow communication with the caudal maxillary sinus (CMS). If drainage is impaired, residual infection may persist locally despite the resolution of the original underlying condition.

Interestingly, the second clinical case included in this study demonstrates that the MCS may also be involved in chronic primary sinusitis not related to dental pathology. In this case, the sinusitis presented with marked changes in the nasal cavity and paranasal sinuses, as revealed by CT, suggesting a possible fungal involvement. These alterations may have impaired normal sinus drainage, potentially explaining the secondary involvement of the MCS despite the absence of dental disease. Overall, these observations highlight that the MCS is a clinically relevant sinus compartment that should not be overlooked in cases of chronic or recurrent sinusitis.

The main limitations of this study are the limited sample size, which may limit the interpretation of the results, particularly given the potential impact of individual anatomical variability. Then, the radiographic images were reviewed by a non‐blinded observer who was aware of the location of the markers. This could have introduced a degree of observational bias in interpretation. Finally, no CT‐based volumetric assessment of the MCS was performed across the different heads, limiting conclusions regarding size variations in both healthy and affected horses.

## 5 CONCLUSION

The margins of the MCS are difficult to define on standard radiographs of cadaver heads from horses without evidence of sinonasal disease. However, consistent landmarks—particularly in LL projections—can be used to approximate its location in adult horses, using the ethmoturbinates as a reliable point of reference. This was supported by comparison with two clinical cases of MCS sinusitis, confirmed by CT and clearly identifiable on radiographs.

## FUNDING INFORMATION

Not applicable.

## CONFLICT OF INTEREST STATEMENT

The authors declare no conflicts of interest.

## AUTHOR CONTRIBUTIONS

**J.‐B. Pavard:** Conceptualization; writing – original draft; resources; project administration; writing – review and editing; investigation; methodology. **A. Young:** Methodology; validation; supervision. **M. Robert:** Methodology; validation; supervision; investigation. **D. Verwilghen:** Conceptualization; investigation; methodology; validation; supervision; project administration.

## DATA INTEGRITY STATEMENT

Jean‐Baptiste Pavard had full access to all the data in the study and takes responsibility for the integrity of the data and the accuracy of data analysis.

## ETHICAL ANIMAL RESEARCH

Research ethics committee oversight was not required for this study, as it was performed on material collected during post‐mortem examination.

## INFORMED CONSENT

Explicit owner consent for inclusion of animals in this study was not obtained. All owners, trainers, or agents were informed that data and tissues might be used for research in general, and verbal consent was obtained at the time of euthanasia.

## Data Availability

The data that support the findings of this study are openly available in Zenodo at https://zenodo.org/records/20546247?preview=1&token=eyJhbGciOiJIUzUxMiJ9.eyJpZCI6ImExN2RjN2JmLTIxMmMtNDFkZS1hNWY0LTA3NTBkZWM0NDhkMCIsImRhdGEiOnt9LCJyYW5kb20iOiIzNTY0ZTYxYTcxMDRmOTZjZTdlODVhY2ExZTY3MGM2YyJ9.9KlkOsw6cnfVQzfanxuKj36DqJJf9rtnXzFC7WiCs0a_TkAo2YYJZJu_ah2T73TDIrUjhcjnGxWq1dt9yWr4pg.
